# Editorial for Special Issue “Advances in Molecular Therapies and Disease Associations in Diabetes”

**DOI:** 10.3390/cimb48101072

**Published:** 2026-10-17

**Authors:** Hongbao Cao, Shaolei Teng

**Affiliations:** 1 School of Systems Biology, George Mason University, Fairfax, VA 22030, USA; 2 Department of Biology, Howard University, Washington, DC 20059, USA; shaolei.teng@howard.edu

Diabetes research increasingly connects metabolic dysfunction with the molecular processes that shape injury across organs. Mechanistic work linking hyperglycemia, oxidative stress, and vascular damage helped establish this perspective [1]. The Special Issue, “Advances in Molecular Therapies and Disease Associations in Diabetes”, extends it through five contributions addressing oxidative stress, cardiovascular remodeling, perioperative inflammation, and renal dysfunction. Its central value is to connect therapeutic possibilities with clinically observable complications, supporting research that links molecular intervention to meaningful patient outcomes.

Therapeutic developments illustrate the value of this broader perspective. EMPA-REG OUTCOME established cardiovascular benefits of empagliflozin in patients with type 2 diabetes and high cardiovascular risk [2]. SURPASS-2 demonstrated the efficacy of dual GIP/GLP-1 receptor agonism with tirzepatide for glycemic and weight outcomes against semaglutide 1 mg [3]. More recently, FLOW demonstrated kidney outcome benefits of semaglutide in patients with type 2 diabetes and chronic kidney disease [4]. These advances support attention to organ protection and metabolic treatment together. Questions remain about complication-specific mechanisms, variation in treatment response, and the added value of molecular biomarkers. The contributions in this collection approach these questions from complementary perspectives.

Grigoriou and colleagues examine diabetes-associated atrial fibrillation through the interacting effects of metabolic remodeling, inflammation, oxidative stress, fibrosis, mitochondrial dysfunction, and altered electrophysiology [5]. Their review connects these mechanisms to emerging strategies involving inflammatory pathways, atrial ion channels, and mitochondrial function, while discussing the potential relevance of SGLT2 inhibitors and GLP-1 receptor agonists. This contribution offers a framework for studying the atrial substrate underlying arrhythmia and for relating metabolic treatment to rhythm-control research. It also emphasizes the need for clinical validation: plausible antiarrhythmic mechanisms and encouraging findings do not establish equivalent benefits across drug classes, patient groups, or rhythm-control endpoints.

Dinu (Iacob) and colleagues consider the relationship between antidiabetic drug development and oxidative stress by reviewing synthetic sulfonamides and natural antioxidants [6]. Their synthesis highlights the rationale for compounds or combinations that address metabolic abnormalities together with redox-related injury. The contribution broadens the therapeutic discussion by placing chemical design and antioxidant activity within a common research framework. The proposed synergy is best viewed as a direction for investigation, requiring direct comparison with individual components, relevant dose–response studies, and assessment of pharmacokinetics and safety before clinical benefit can be established.

The original study by Rakoczy and colleagues adds a clinical perspective on profibrotic signaling by investigating circulating transforming growth factor beta 1 (TGF-β1) in individuals with early coronary artery disease, including those with type 2 diabetes [7]. Reported associations with ventricular function were weak, and TGF-β1 was not significantly associated with diabetes or several other conventional cardiovascular risk factors. These findings help refine the interpretation of a biologically relevant mediator: its involvement in tissue remodeling does not automatically make its circulating concentration a diabetes-specific marker. The study motivates closer integration of molecular measurements with cardiac phenotyping and independent evaluation of their diagnostic or prognostic value.

Stougiannou and colleagues extend the cardiovascular focus to cardiac surgery with cardiopulmonary bypass [8]. Their review considers how diabetes-related inflammatory and metabolic disturbances intersect with the inflammatory response associated with bypass. By bringing these processes into a shared pathophysiological framework, the article draws attention to the perioperative setting as an opportunity to investigate susceptibility to organ injury. It encourages research linking preoperative metabolic status, perioperative molecular responses, and postoperative outcomes, with careful separation of mechanistic proposals from interventions supported by clinical trials.

Khandker and colleagues address renal complications through a retrospective case–control study of serum creatinine, urea, and glomerular filtration rate [9]. The diabetic group showed higher creatinine and urea levels and lower filtration rate, reinforcing the clinical relevance of accessible measures of renal impairment. This contribution complements the molecular themes of the collection by anchoring disease associations in routine laboratory assessment. Its findings support further investigation of the relationship between metabolic status and kidney function, while longitudinal studies are needed to determine temporal progression and predictive performance. Future molecular biomarkers should be evaluated for the information they add to established renal assessments.

Viewed together, these contributions connect therapeutic discovery with the characterization of organ dysfunction. They also identify a practical translational challenge: the same broad processes, such as inflammation or oxidative injury, may contribute to several complications without having identical effects in every tissue or disease stage. We therefore see particular value in research that specifies where a pathway acts, when it becomes important, and which patients are most likely to benefit from its modulation. The collection provides useful starting points for this work by linking candidate interventions to cardiovascular, surgical, and renal contexts.

Several priorities follow from this synthesis. Longitudinal cohorts should combine repeated molecular measurements with clearly defined clinical outcomes to distinguish markers of existing injury from predictors of future deterioration. Biomarker studies should test incremental value over standard clinical models, use independent validation populations, and examine whether the findings could change a management decision. For therapeutic development, experiments should establish target engagement and the direction of effect in relevant tissues, followed by trials that measure organ-specific outcomes and adverse events. Combination strategies require evidence of benefit beyond their individual components.

Patient heterogeneity is another priority. Data-driven subgrouping of adult-onset diabetes has identified groups with differing risks of complications [10], providing a rationale for more detailed biological characterization. Integrating multi-omics data with imaging and routine laboratory measurements could help identify mechanisms of injury or treatment response within such groups. Studies should use reproducible workflows, transparent evidence-based network and pathway interpretation, and validation across populations. Disease duration, medication exposure, renal function, sex, and ancestry should be considered so that molecular differences can be interpreted in their clinical context. The aim should be a testable explanation of disease variation that supports a specific clinical or experimental decision.

As this Special Issue closes, its contributions underscore the value of studying diabetes through the molecular links between metabolic dysfunction, therapeutic action, and associated disease. The collection offers mechanistic frameworks, candidate therapeutic directions, and clinical observations that can guide the next stage of investigation. Progress will depend on connecting these insights to validated biomarkers and interventions that improve outcomes for patients with diabetes and its complications. We thank the contributing authors, reviewers, and the editorial team of *Current Issues in Molecular Biology* for their work in developing this Special Issue.
